# Predictive Dose-Based Estimation of Systemic Exposure Multiples in Mouse and Monkey Relative to Human for Antisense Oligonucleotides With 2′-*O*-(2-Methoxyethyl) Modifications

**DOI:** 10.1038/mtna.2014.69

**Published:** 2015-01-20

**Authors:** Rosie Z Yu, John S Grundy, Scott P Henry, Tae-Won Kim, Daniel A Norris, Jennifer Burkey, Yanfeng Wang, Andrew Vick, Richard S Geary

**Affiliations:** 1 Preclinical and Clinical Development, Isis Pharmaceuticals, Inc., Carlsbad, California, USA; 2 Analytical Services, WIL Research Laboratories, Ashland, Ohio, USA

## Abstract

Evaluation of species differences and systemic exposure multiples (or ratios) in toxicological animal species versus human is an ongoing exercise during the course of drug development. The systemic exposure ratios are best estimated by directly comparing area under the plasma concentration-time curves (AUCs), and sometimes by comparing the dose administered, with the dose being adjusted either by body surface area (BSA) or body weight (BW). In this study, the association between AUC ratio and the administered dose ratio from animals to human were studied using a retrospective data-driven approach. The dataset included nine antisense oligonucleotides (ASOs) with 2′-*O*-(2-methoxyethyl) modifications, evaluated in two animal species (mouse and monkey) following single and repeated parenteral administrations. We found that plasma AUCs were similar between ASOs within the same species, and are predictable to human exposure using a single animal species, either mouse or monkey. Between monkey and human, the plasma exposure ratio can be predicted directly based on BW-adjusted dose ratios, whereas between mouse and human, the exposure ratio would be nearly fivefold lower in mouse compared to human based on BW-adjusted dose values. Thus, multiplying a factor of 5 for the mouse BW-adjusted dose would likely provide a reasonable AUC exposure estimate in human at steady-state.

## Introduction

**Figure 1:**
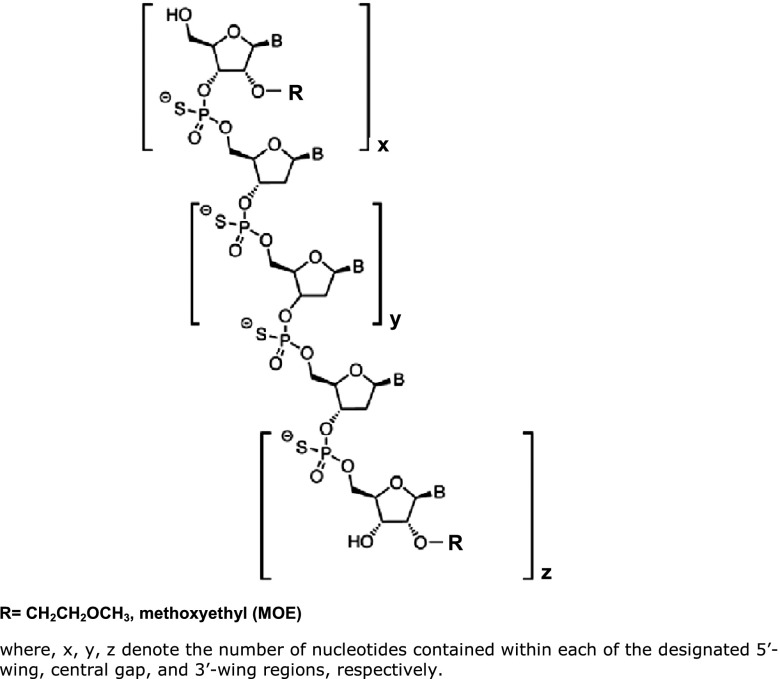
**General structure of 2′-MOE modified ASOs with a chimeric (gapmer) design.**

Antisense oligonucleotides (ASOs) with 2′-*O*-(2-methoxyethyl) (2′-MOE) (**Figure 1**), represent a platform of RNA-based therapeutics designed to specifically hybridize to their target RNA via Watson-Crick base pairing and prevent expression of the encoded “disease-related” protein product. The last decade has seen a very rapid increase in the number of 2′-MOE ASOs progressing to phase 1, 2, and 3 clinical trials and targeting ever expanding therapeutic areas of interest including, but certainly not limited to, rheumatoid arthritis,^1,2^ diabetes,^3^ cancer,^4,5^ hypercholestrolemia,^6,7,8^ and multiple sclerosis.^9^ A 2′-MOE ASO, mipomersen (Kynamro), was recently approved by the US FDA as an adjunct to lipid-lowering medications and diet to reduce atherogenic lipids in patients with homozygous familial hypercholesterolemia (HoFH).

Determination of systemic (plasma) exposure ratios in toxicological animal species versus humans are best done by comparing area under the plasma concentration-time curve (AUC) values. Such exposure ratios are commonly used to relate the exposure achieved in animal pharmacology or toxicology studies to human and thus, facilitate the assessment of the relevance of these findings to clinical efficacy or safety.^10^ For example, determination of the “margin of safety” or “margin of exposure” is typically done based on the plasma AUC ratio of the no observable adverse effect level (NOAEL) or the lowest observable adverse effect level (LOAEL) in animals to the observed exposure in humans at the dose levels intended for clinical use. Understanding the PK and exposure differences between species would help to define the safety margins and human dose selections.

Details of preclinical and clinical pharmacokinetic properties and interspecies scaling of several 2′-MOE ASOs, have been reported previously.^11,12^ An article describing some initial evaluations of the predictive performance of several different interspecies scaling approaches for ASOs was recently published.^13^ In the case of 2′-MOE ASOs, the most common animal species tested are mice and monkeys. Therefore, it is an important question to ask what dose-adjusted comparisons between these toxicology species and human best estimate the relative systemic exposure ratio. Does the same dose-adjusted scaling approach work well for both species when extrapolating to human, or are species-dependent scaling approaches needed since mouse and monkey may perform differently when scaling to man as reported previously?^11^

In this article, the association between relative systemic exposure (plasma AUC) and dose (adjusted by either body surface area (BSA) or body weight (BW)) from animals (mice and monkeys) to human for nine 2′-MOE ASOs was studied using a retrospective data-driven approach. These nine selected ASOs, with 20 or 21 nucleotides in length, have similar physicochemical properties, including charge, molecular weight, and amphipathic nature, and share similar pharmacokinetic characteristics such as comparable protein binding and tissue distribution.^11,12,17^ This class of ASOs all have the same chemical modifications on the backbone structure and the sugar moiety (phosphorothioate and 2′-MOE, respectively), thus have prolonged in vivo half-lives due to increased nuclease resistance and metabolic stability in animals and humans.^11,12,18^ Since ASOs are extensively distributed to tissues, where are often considered as the site(s) of actions for both pharmacologic and toxicologic activities, the plasma exposure ratio and the liver exposure ratio were compared between animal species. The consistency between plasma exposure ratio and tissue exposure ratio would support the use of plasma AUC exposure ratio, instead of tissue ratio, to guide the selection of dose-based estimation of systemic tissue exposure in humans.

## Results

### Pharmacokinetic properties of ASOs

The primary route of administration for oligonucleotides for systemic applications is by parenteral injection, either intravenous (i.v.) infusion or subcutaneous (s.c.) injection. Following systemic s.c. or i.v. administration, plasma ASO concentrations rapidly declined from peak concentrations in a multiexponential fashion as characterized by a dominant initial rapid distribution phase (half-life of a few hours or less) representing extensive distribution to tissues, followed by a much slower terminal elimination phase (half-life of 2–4 weeks) in both animals and humans as reported previously.^11,12,14^ The apparent terminal elimination rate observed in plasma is consistent with the slow elimination of ASOs from tissues, indicating equilibrium between postdistribution phase plasma concentrations and tissue concentrations.^14^

There is little or no accumulation in plasma AUC (or *C*_max_) values upon every other day or once weekly repeated dosing in both monkeys and humans. Mean plasma AUCs following both single and multiple dosing of a 2′-MOE ASO are generally comparable, with “steady-state” in plasma being essentially achieved with the very first dose in both monkeys and humans. In mice, plasma AUC did increase after repeated administrations (although little change in *C*_max_, data not shown), likely resulted from saturation of kidney uptake in this species.^11,12,14^ The ASOs with TK data showed similar dose-normalized exposure within the same species with relatively low variability (%CV in the range of 29–38%).

In this article, we employed the PK properties to compare the dose-normalized AUC data for multiple 2′-MOE ASOs across species as a measure of scaling between exposure multiples. The mean dose-normalized AUC ratios at steady-state were 2.58, 15.7, and 14.1 to 18.3 in mice, monkeys, and humans, respectively (**Tables 1** and **2**). Therefore, dose-normalized exposure in mice was substantially different from monkey and human, while it was very similar between monkeys and humans.

### Relative ratios of mouse to human

**Figure 2:**
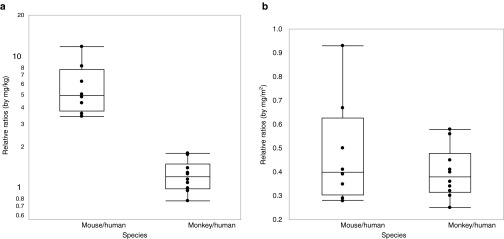
Box plot of calculated relative ratios between mouse/human and monkey/human based on (**a**) dose adjusted for body weight (mg/kg) and (**b**) dose adjusted for body surface area (mg/m^2^) following multiple dose administrations.

As shown in **Table 1**, the systemic plasma exposure ratios (PER) varied substantially among ASOs, so were the BW- or BSA-normalized administered dose ratios (ADR), which were not surprising since different dose levels were used in the toxicology studies. The relative ratios (RR) (mean ± SD) between mouse and human was 0.82 ± 0.35 for single doses and 0.48 ± 0.22 when dose was adjusted for BSA following multiple doses (**Table 1**, **Figure 2**). The RR (mean ± SD) was 10.1 ± 4.3 and 5.87 ± 2.75 when dose was adjusted for BW following single and multiple doses, respectively.

Following repeated doses, the BSA-adjusted dose ratio would under-predict the AUC ratios at steady-state by ~50%. On the other hand, the BW-adjusted dose ratios would over-predict the AUC ratios for both single and multiple doses, by approximately ten- and fivefold, respectively. Taken together, these data suggest that neither the BSA-adjusted nor BW-adjusted dose ratios can directly predict the AUC ratio at steady-state between mice and humans for 2′-MOE ASOs. However, considering the similarity of ASOs within the same species, the AUC ratios might be predicted by BW- or BSA-adjusted dose ratios if corrected by certain factors. For example, the BW-adjusted dose ratio from mouse to human divided by a factor of 5 or the BSA-adjusted dose ratio multiplied by a factor of 2 would provide a reasonable estimate for the steady-state AUC ratio.

### RR monkey to human

For monkey to human comparisons, the RR (mean ± SD) between monkey and human was only 0.33 ± 0.12 for single dose and 0.39 ± 0.11 for multiple doses when dose was adjusted for BSA suggesting that BSA-adjusted dose ratio cannot be used to predict plasma exposures directly following both single and multiple doses (**Table 2**, and **Figure 2**). Nonetheless, unlike predictions from mouse to human, similar RRs were obtained following multiple doses as compared to single dose for monkey to human.

However, when dose was adjusted for BW, the RR was 1.02 ± 0.38 for single dose and 1.21 ± 0.35 for multiple doses when dose was adjusted for BW (**Table 2**, and **Figure 2**). Taken together, these data suggest plasma AUC ratio between monkeys and humans for 2′-MOE ASOs can be predicted by the BW-adjusted dose ratio following both single and multiple doses.

### Comparison of RR between plasma AUC and liver concentration

Liver contains high concentrations of oligonucleotides following parenteral administrations and is the primary organ of oligonucleotide distribution due to its large size.^11,12,14^ For this reason, liver has been the primary therapeutic target for majority of antisense oligonucleotides currently in development.

In this study, where both plasma AUC and tissue concentrations were available in animals, the relative exposure ratios were compared between rodent species or between rodent and monkey. As shown in **Table 3**, the ratio between the species was the same for either exposure measure, indicating that plasma AUC ratios can be used to estimate the relative tissue exposure between species.

## Discussion

The results of this retrospective analysis indicate that, for 2′-MOE ASOs, the proper plasma AUC scaling factors are different for mouse and monkey. As an example, comparable AUC values would be expected in monkey and human at equivalent mg/kg dose levels, while the plasma exposures in mouse would be nearly fivefold lower at steady-state (**Tables 1** and **2**). Thus, an empirical value of fivefold of the dose ratio after adjusting the dose by BW following multiple doses can probably be used to estimate systemic exposure between mice to human following multiple doses (or at steady-state). Although the data are limited, widened gap sizes instead of the standard 5-10-5 construct in two of the studied ASOs (4-13-4 for OGX-011 and 2-16-2 for ISIS 325568) did not appear to affect the exposure and calculated RR between mouse and monkey to human.

The results presented here also appear generally consistent with some previously published literature^11,12,14^ describing allometric scaling of plasma clearance versus BW for 2′-MOE ASOs. Plasma clearance is inversely related to plasma AUC (*i.e.*, CL = Dose/AUC). Geary *et al*. reported a general simple linear allometric relationship of plasma clearance versus BW with a slope of ~1.0 for ISIS 104838 (a 2′-MOE ASO) across rat, monkey, dog, and human, but mouse was an “outlier” and thus was excluded from this relationship. This analysis supports BW based dose scaling from rat to human. Whereas, Yu *et al*. reported an attempt to develop a simple allometric relationship of plasma clearance versus BW across all evaluated species, including mouse, rat, monkey, and human for ISIS 301012 (mipomersen; Kynamro). This analysis generated an allometric exponent (slope) of 0.6461, suggesting BSA based dose scaling, which has an allometric exponent of 0.67. It is also worth noting that the regression line from the Yu *et al*. publication appears to better fit observed mean mouse and human mean plasma clearance data compared to monkey.

Mahmood^13^ also attempted to predict previously published observed mean human plasma clearance values for phosphorothioate oligodeoxynucleotides and 2′-MOE ASOs, utilizing previously published mean clearance data of the same compounds from several tested animal species, including mouse, rat, dog, and monkey, and applying various allometric scaling approaches. In this publication, human plasma clearance predictions were based on scaling data from one, two, or three animal species and the findings demonstrated mixed success. Mahmood reported that allometric scaling based on one or two species can be “erratic and unreliable,” although either fixed exponent or fixed coefficient approaches were evaluated for the one species allometric evaluations. Scaling approaches based on BSA or BW as described here in our article were not included. Further, Mahmood indicated that reasonably accurate predictions could be obtained using at least three animal species; albeit the reported ratios of predicted to observed human clearance values for four different ASOs (two first generation and two second generation compounds) appear highly variable and ranged from 0.05 to 1.29. Only two of the four evaluated ASOs from the three species scaling were within the acceptable prediction range, with none of the predictions for the other two ASOs being within the acceptable range of 0.5–2.0.

**Figure 3:**
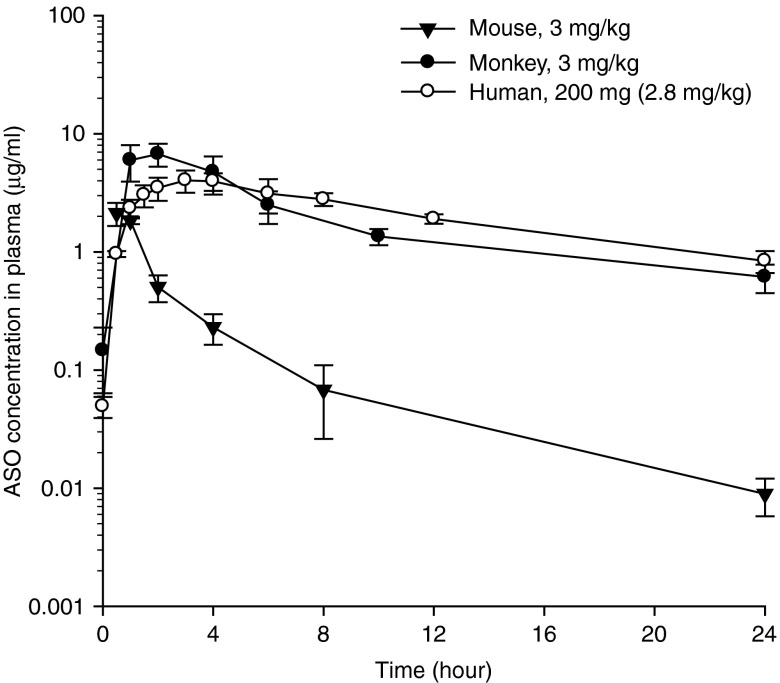
**Comparative plasma concentration-time profiles of a 2′-MOE modified ASO in mouse, monkey and human at equivalent mg/kg dose levels.**

It is our opinion that simple allometric scaling approaches for 2′-MOE ASOs that utilize multiple species are likely of limited value and can provide misleading results since mouse is often included in the scaling analysis. The reasons for the mouse being an “outlier” could probably be due to the special physiology and anatomy of the mouse animal model and the special PK characteristics of ASOs. Mouse seems to have an exceptionally large liver and kidneys relative to its BW, with liver and kidney weight being nearly threefold higher relative to monkey and human.^15^ The difference in liver and kidney size (relative to the BW) could be translated into substantial PK differences for ASOs since all known 2′-MOE ASOs are highly distributed into liver and kidney tissues, with liver and kidney concentrations being ~5,000- and 8,000-fold higher concentration over plasma trough levels based on data from literatures.^16,17^ Liver and kidneys are not only a distribution organ but also an elimination organ since ASOs are generally metabolized by endonuclease in the tissues including liver and kidneys. Thus, a relatively large liver and kidneys in the mouse means both a higher clearance and higher volume of distribution for ASOs, leading to a lower plasma AUC but a similar terminal half-life in the mouse, as shown in **Figure 3**. Perhaps somewhat more complex allometric scaling models that include multiple factors such as species organ weights/volumes, plasma protein binding, etc., are worth further evaluation and may allow better predictions across multiple species.

Common simple allometric scaling approaches inherently assume “more species are better.” Such an approach will often reasonably well apply across multiple species and ultimately lead to more accurate human clearance predictions. While these approaches may indeed be suitable for many small molecule compounds, we would argue otherwise based on our current investigations for 2′-MOE ASOs as discussed above. Acceptable human plasma clearance predictions can be made based on just a single species (mouse or monkey), after appropriate application of a species-specific scaling approach. It is also worth noting that acceptable human plasma exposure predictions for a new clinically untested 2′-MOE ASO can be reasonably estimated based on past clinical experience with other 2′-MOE ASOs.^18^

Our findings are established based on dosing, pharmacokinetic and exposure data in multiple species from nine different 2′-MOE ASOs. This type of analysis was made possible given the remarkable similarity in the pharmacokinetic properties of 2′-MOE ASOs from sequence to sequence within species, which has been reported previously.^11,12,14,18^ Furthermore, this translates to remarkable similarity in how these types of compounds, as a class, scale from mouse to human and from monkey to human. Nonetheless, our findings suggest that there is not a simple common dose scaling approach applicable between all three species (*i.e.*, mouse, monkey, and human).

In conclusion, the results of this retrospective analysis indicate that, for 2′-MOE ASOs, the proper scaling factors are different for mouse and monkey. Between monkey and human, the plasma exposure ratio can be predicted directly based on BW-adjusted dose ratios, while between mouse and human, the steady-state exposure ratio would be nearly fivefold lower based on BW-adjusted dose values. Thus, multiplying a factor of 5 for the mouse dose would likely provide a reasonable AUC exposure estimate in humans at steady-state. The assumption and relationship can be further validated as the database continues to grow steadily as more compounds enter development.

## Materials and methods

*Test compounds.* Retrospective preclinical and clinical study data (from either published sources or available internally at Isis Pharmaceuticals) from a total of nine ASOs, which share a similar chemical composition, 20 or 21 nucleotides in length, were evaluated (**Table 4**). The 2′-MOE ASOs are phosphorothioate oligonucleotides containing 2′-MOE sugar modifications on the 3′- and 5′-ends (“wings”) of the molecule that flank a central DNA-like region (“gap”), and thus utilize a chimeric design strategy (*i.e.*, the wings provide increased affinity and nuclease resistance, whereas the central gap allows RNase H-mediated cleavage of the target “sense” RNA) (**Figure 1**).

*Dose conversions.* In humans, clinical doses (typically given as fixed mg doses) were converted to mg/kg levels based on an assumption of BW of 70 kg, as needed. In animals, the doses were generally given as mg/kg. In both animals and humans, conversion of mg/kg to mg/m^2^ dose levels were made based on well-accepted conversion factors, *i.e.*, mg/kg dose multiplier values of 3, 12, and 37 for mouse, monkey, and human, respectively, to determine corresponding mg/m^2^ dose.^19^

*Mouse toxicology/toxicokinetic studies.* Single and multiple dose toxicology /toxicokinetic (TK) studies were conducted in male and female CD-1 mice (Crl:CD-1 (ICR) BR; Charles River Laboratories, Wilmington, MA). Two dose levels were generally tested per compound, ranging from 3 to 40 mg/kg (9–120 mg/m^2^) administered by s.c. injection. ASOs were administered every other day for four doses (as loading dose for one week), followed by dosing once every fourth day or once a week for the remainder of a 4- to 13-week dosing period. Blood samples were collected for ASO quantitation in plasma by cardiac puncture at sacrifice in tubes containing EDTA at various time points over a 48-hour period following the dose (three mice per time point), and plasma was harvested. Both single and multiple dose plasma exposure data (mean AUCs) were used respectively for systemic exposure multiple determinations. In addition, liver and kidney samples were collected for drug concentration analysis at sacrifice ~48 hours after the last dose. In this study, only liver exposure data were included and compared across species.

*Monkey toxicology/toxicokinetic studies.* Single and multiple dose toxicology/toxicokinetic studies were conducted in male and female cynomolgus monkeys (*Macaca fascicularis*; Sierra Biomedical Animal Colony, Sparks, NV). Four dose levels ranging from 1 to 40 mg/kg were generally tested for each compound administered via 1-hour i.v. infusion or s.c. injection. One of the four doses selected, ranging from 2 to 4 mg/kg (24–48 mg/m^2^), close to the clinical dose, was selected and included in this analysis. ASOs were administered every other day for four doses (as loading dose for one week), followed by dosing once every fourth day or once a week for the remainder of a 4- to 13-week dosing period. Blood was collected for quantitation of oligonucleotide concentrations in plasma by peripheral venipuncture into EDTA containing vacutainers at various time points over a 48-hour period following the dose during treatment as well as post treatment period, and plasma was harvested. Both single and multiple dose plasma exposure data (mean AUCs) were used respectively for systemic exposure multiple determinations. In addition, liver and kidney cortex samples were collected for drug concentration analysis at sacrifice ~48 hours after the last dose. In this study, only liver exposure data were included and compared across species.

All mouse and monkey studies were conducted utilizing protocols and methods approved by the Institutional Animal Care and Use Committee (IACUC) and carried out in accordance with the Guide for the Care and Use of Laboratory Animals adopted and promulgated by the US National Institutes of Health.

*Human (clinical) studies.* Human data were mostly from phase 1 clinical studies conducted in healthy volunteers or cancer patients. ASOs were dosed as 2-hour i.v. infusion or s.c. injection at dose levels that ranged from 175 to 640 mg (2.5–9.14 mg/kg; 92.5–338 mg/m^2^). Doses were administered on days 1, 3, 5, and 8 as a loading regimen, followed thereafter by once weekly administrations for an additional 3–5 weeks. Intensive pharmacokinetic blood sampling at various time points occurred for 24 or 48 hours following an i.v. or s.c. dose. Samples were collected in EDTA tubes and plasma was harvested. Single and multiple dose plasma exposure data (mean AUCs) were used for systemic exposure multiple determinations.

*Analytical methods.* Plasma samples were analyzed for parent ASO concentrations using quantitative and sensitive hybridization ELISA methods which were a variation of a previously reported method.^20^ ASO concentrations in tissue samples were quantitated using capillary gel electrophoresis (CGE) or HPLC with UV detection.^12,20^ These assays were validated for precision, accuracy, selectivity, sensitivity, metabolite cross-reactivity, dilution linearity, prozone effect, and stability of parent oligonucleotide prior to analysis of mouse, monkey, and human plasma or tissue samples. Both plasma and tissue sample analyses were conducted based on the principles and requirements described in 21 CFR part 58. The lower limit of quantitation (LLOQ) of the validated assays ranged from 0.2 to 2.0 ng/ml in mouse, monkey, and human plasma, and from 0.2 to 10.0 µg/ml in mouse and monkey tissues.

*Determination of pharmacokinetic plasma exposure (AUC).* The area under the plasma concentration-time (AUC) values in individual animal and human were calculated using the linear trapezoidal rule (WinNonlin 3.1 or higher, Pharsight, Mountainview, CA) and summarized using descriptive statistics. Partial area plasma AUC (AUC_0–24 hours_ or AUC_0–48 hours_) values typically represent >90% of total AUC (AUC_0-∞_ following single dose, or AUC_0–τ_ at steady-state) because the plasma distribution phase dominating plasma exposure and clearance of 2′-MOE ASOs.^18^ While other plasma PK exposure parameters were also typically determined, this retrospective analysis focused on plasma AUC only given that it is the most commonly applied metric to assess systemic exposure multiples.

*Determination of systemic exposure and administered dose ratios.* Systemic plasma exposure ratio (PER) is defined based on the mean plasma AUC values in animals and human at reported doses without adjustment for BW or BSA (Eq. 1). Similarly, the liver exposure ratio (LER) is defined based on reported mean liver concentrations between animal species (no liver tissue data from patients), and the administered dose ratio (ADR) after adjustment for BW (mg/kg) or BSA (mg/m^2^), are defined as shown below:

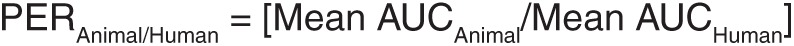


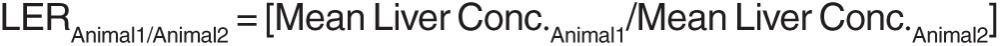


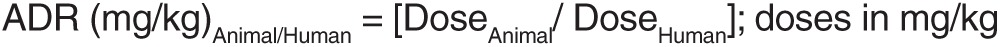


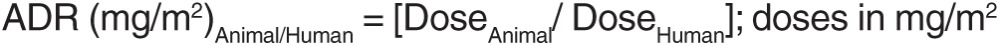


In addition to the equations above, another metric designated as the “relative ratios” (*i.e.*, ratio of PRM/ADR or LER/ADR) is defined to assess how well an administered dose ratio (based on either mg/kg or mg/m^2^ adjustment) estimates the corresponding systemic exposure ratio, with a relative multiple ratio value of 1.0 being a perfect predictive match, and a calculated value between 0.5 and 2.0 considered acceptable. The RR are calculated as follows:

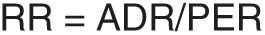


Or

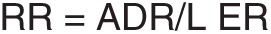


with the ADR calculated based either on mg/kg or mg/m^2^ values.

## Figures and Tables

**Table 1:**
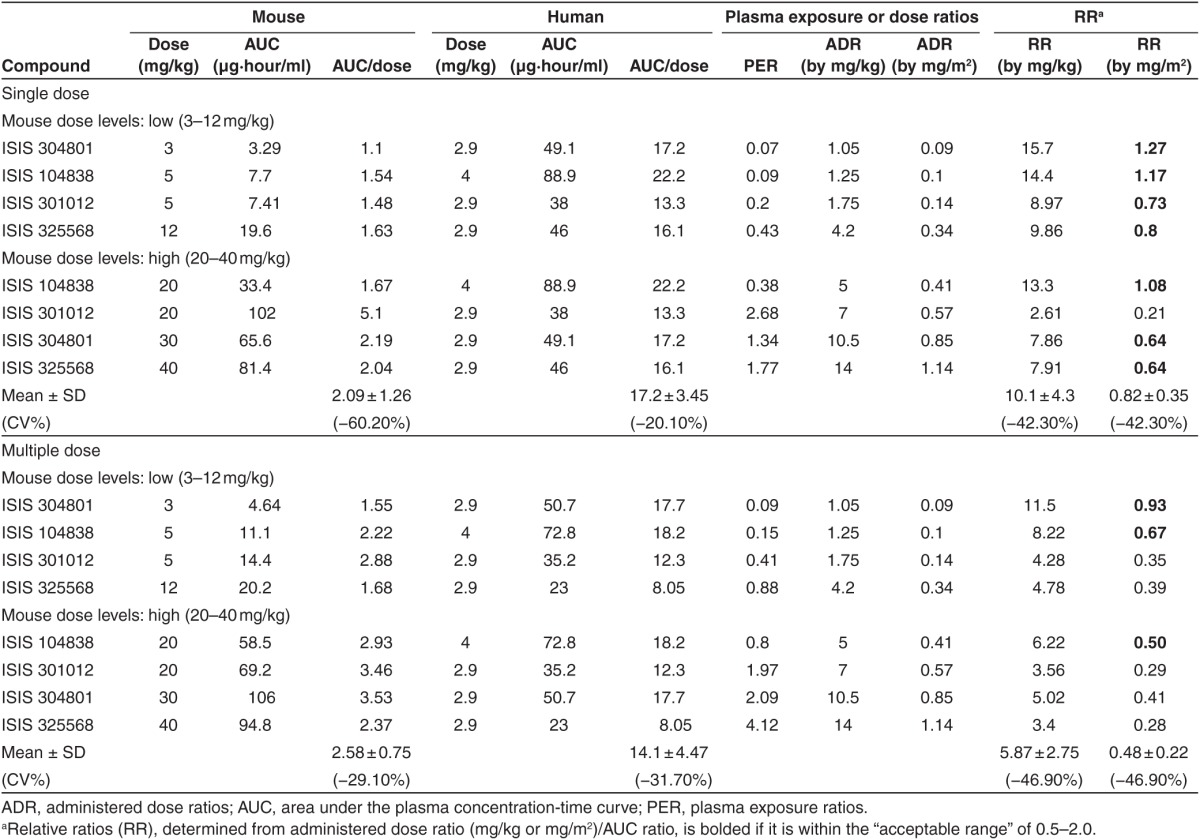
Comparison of exposure, dose, and relative ratios between mouse and human for various second generation antisense oligonucleotides following single dose and multiple doses

**Table 2:**
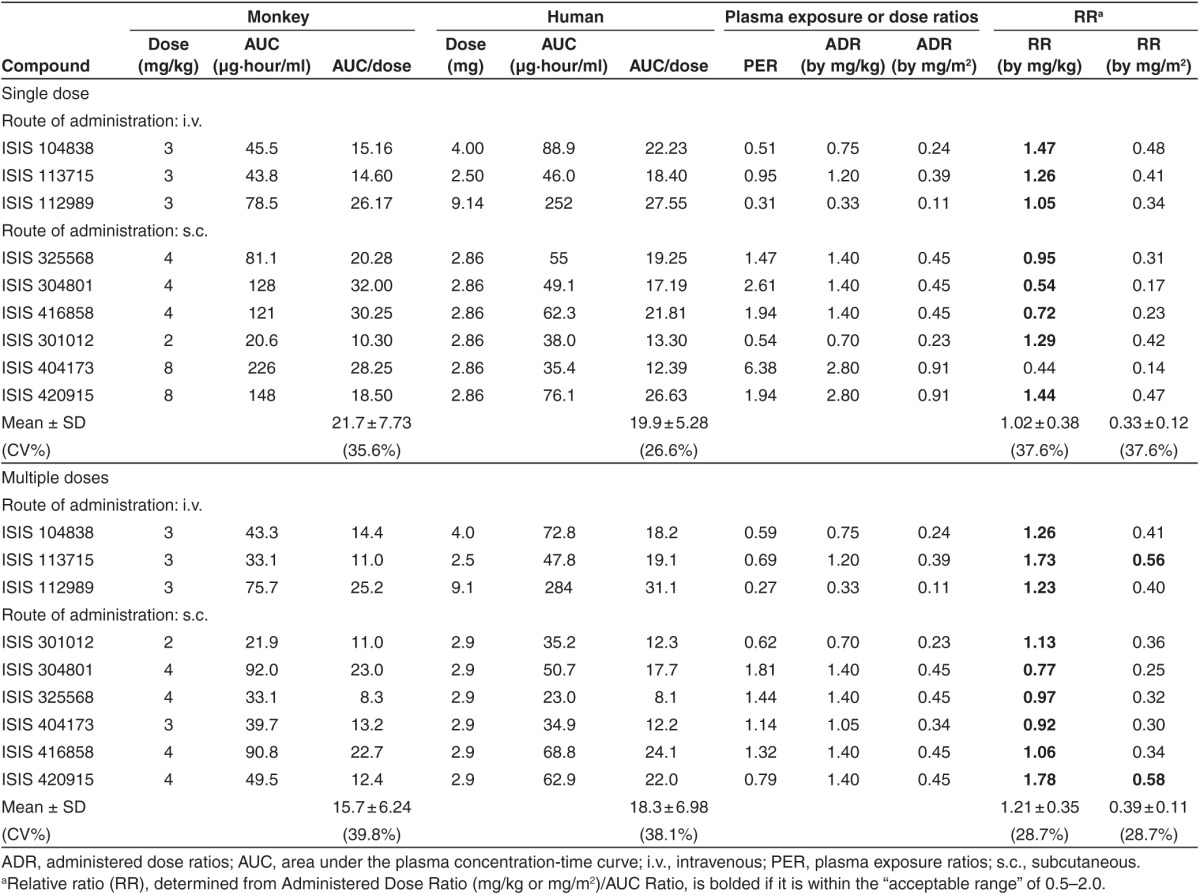
Comparison of exposure, dose, and relative ratios between monkey and human for various second generation antisense oligonucleotides following single dose and multiple doses

**Table 3:**
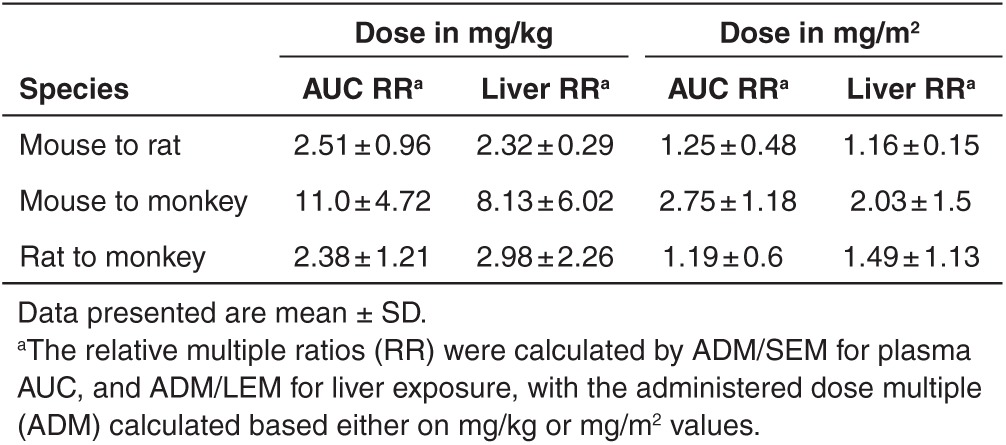
Comparison of relative multiple ratios (RR) calculated based on plasma AUC (SEM) or liver exposure (LEM) between animal species

**Table 4:**
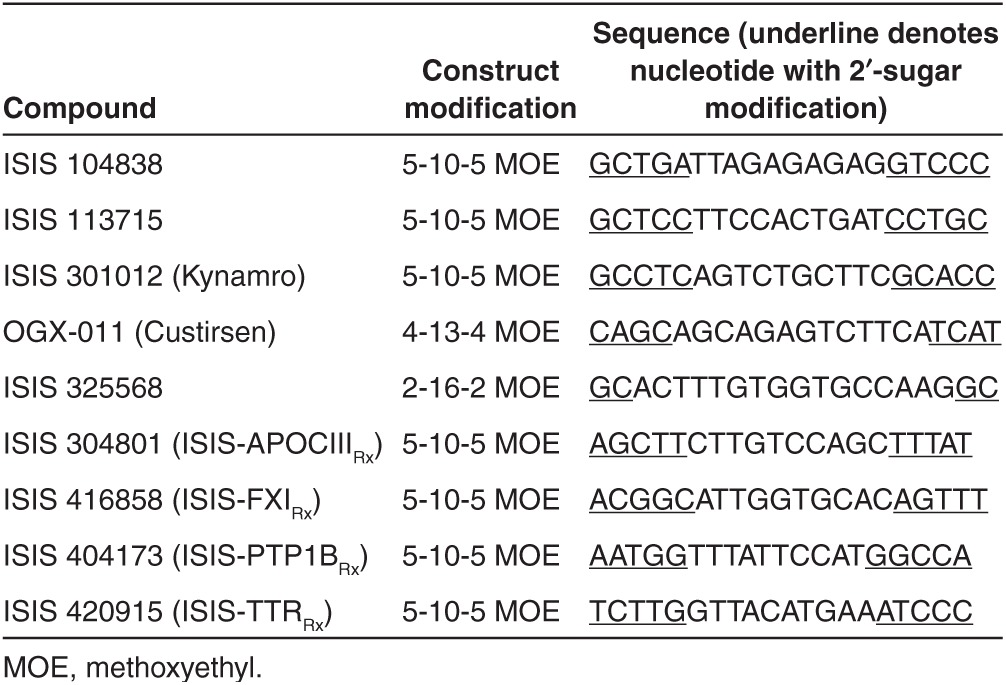
List of second generation ASOs included in the retrospective analysis
